# Unusual Presentation of Left‐Sided Bilothorax Following Spleen Laceration: A Case Without Biliary Tree Injury

**DOI:** 10.1002/ccr3.73841

**Published:** 2026-11-07

**Authors:** Thanuja Reddipilli, Raghava Rao Gandra, Vishwanath Gella, Deekshitha Ithepalli

**Affiliations:** ^1^ Pulmonary Medicine Asian Institute of Gastroenterology Hyderabad India

**Keywords:** blunt trauma, hemoperitoneum, left‐sided bilothorax, spleen laceration, without biliary tree injury

## Abstract

A 46‐year‐old pedestrian sustained blunt injury to abdomen when he was struck from behind by a motorcycle, causing him to fall flat onto the roadway. Ultrasound abdomen showed splenic injury and hemoperitoneum. Chest x‐ray showed left mild pleural effusion. He was managed conservatively by multiple blood transfusions for anemia and referred to our center after 3 weeks. A triphasic CT scan of abdomen revealed a splenic laceration with multiple pseudoaneurysms, moderate hemoperitoneum, and trace bilateral pleural effusions. No biliary tree injury or fistula was identified. On fifth postoperative day after splenectomy, he developed progressive breathlessness and ultrasound showed increased effusion on left side. Around 500 mL of green pleural fluid was aspirated, which was exudative by Light's criteria and cultures were sterile. Pleural fluid to serum bilirubin ratio was more than one, confirming diagnosis of bilothorax. The most likely mechanism for development of bilothorax is due to resorption of hemoperitoneum and splenic hematoma across left diaphragm into pleural space and heme degradation.

## Key Clinical Message

Left‐sided bilothorax may follow splenic trauma without biliary injury via hemoperitoneum resorption across the diaphragm. Pleural effusion after abdominal trauma warrants consideration of this rare diagnosis. Real‐world trauma care requires balancing guidelines with contextual constraints, prioritizing patient safety, structured reassessment, vigilance, and timely drainage to prevent empyema or respiratory failure.

## Graphical abstract

We report a rare case of left‐sided bilothorax in a patient who had spleen laceration and hemoperitoneum after blunt abdominal trauma without any biliary tree injury. A high index of clinical suspicion is important to identify this underdiagnosed entity.

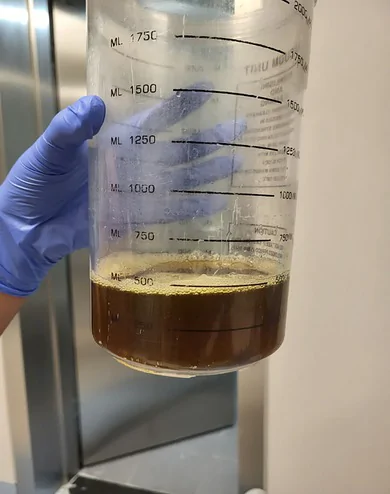

## 1 Introduction

Bilothorax is a rare condition involving the accumulation of bile in the pleural space, with fewer than 120 cases reported. It is thought to occur when negative intrathoracic pressure promotes bile migration through diaphragmatic defects. Bilothorax typically arises secondary to hepatobiliary pathology like biliary obstruction, extension of hepatic infections, subphrenic abscess rupture, diaphragmatic trauma, and iatrogenic injuries from hepatobiliary drain placement [1]. Trauma is the leading cause of noniatrogenic injuries to the biliary system, which can result from penetrating injuries, such as gunshot wounds or stab wounds, or from blunt abdominal trauma, including motorcycle accident [2]. It is one of the rare causes of pleural effusion of extravascular origin. Left‐sided bilothorax is extremely rare. Our case is unique as left‐sided bilothorax occurred due to spleen laceration and resorption of splenic hematoma and hemoperitoneum, without any underlying biliary tree injury.

## 2 Case History

A 46‐year‐old male pedestrian sustained blunt abdominal trauma following a road traffic accident. The mechanism of injury involved being struck from behind by a motorcycle, which precipitated a forward fall onto the roadway surface, resulting in direct abdominal impact. He was immediately managed conservatively elsewhere, where ultrasonography of abdomen showed splenic injury, moderate hemoperitoneum and left mild pleural effusion with partial collapse of basal segments (Figure 1). A chest x‐ray showed a mild left‐sided pleural effusion with no rib fractures. The patient received multiple blood transfusions for correction of anemia and was empirically treated with intravenous amoxicillin–clavulanate for 1 week, prior to referral to our center 3 weeks following the road traffic accident. Given persistent pain in abdomen and drop in hemoglobin, Triphasic Computerized Tomography (CT) scan of abdomen was done which showed Grade 4 splenic laceration with subcapsular hypodensities, multiple splenic pseudoaneurysms involving splenic artery and its branches, high density ascites suggestive of moderate hemoperitoneum and trace bilateral pleural effusions, with no evidence of biliary tree injury or obstruction, or abnormal communication between pleural space and biliary tree (Figure 2). Liver was normal with no intrahepatic biliary duct dilatation or focal lesions or hepatic capsular disruption. Common bile duct was normal in caliber. Gall bladder wall thickness was normal with no evidence of intraluminal calculi or pericholecystic fluid. Pancreas was normal in size, shape and attenuation, with no evidence of ductal dilatation or parenchymal calcifications or peripancreatic fluid collection. Following initial stabilization with transfusions and supportive care, the patient and family were counseled regarding therapeutic options, with emphasis on spleen‐preserving strategies such as selective splenic artery embolization, which are generally preferred over splenectomy. However, financial constraints ultimately led to elective splenectomy 1 month post‐trauma reflecting real‐world practice in many settings. In the absence of such constraints, spleen‐preserving alternatives could have been considered. Adapting management to local realities while prioritizing patient safety remains an important clinical lesson. Intra operative findings during laparotomy revealed lacerated spleen, large splenic hematoma, mild hemoperitoneum, and adhesiolysis of fundus of stomach and colon to spleen was done. No additional intra‐abdominal injuries or visible diaphragmatic lesions were identified.

**FIGURE 1:**
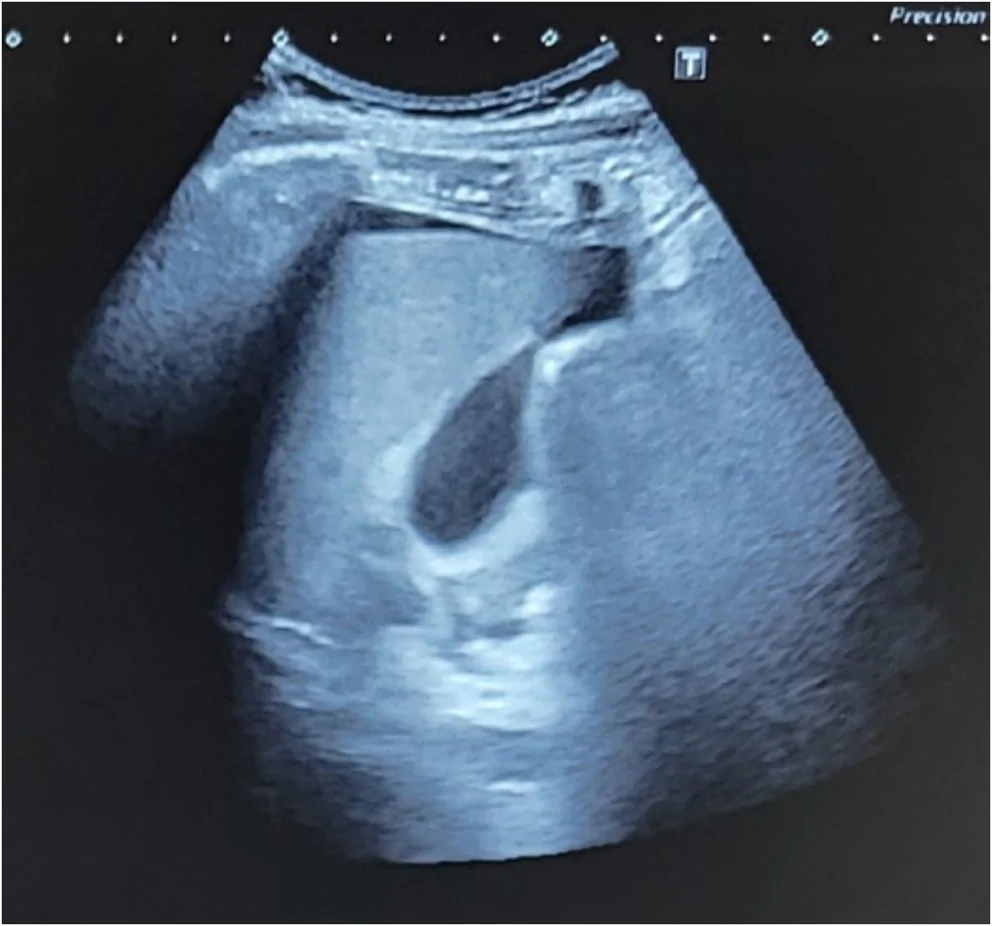
Ultrasound showing left mild pleural effusion.

**FIGURE 2:**
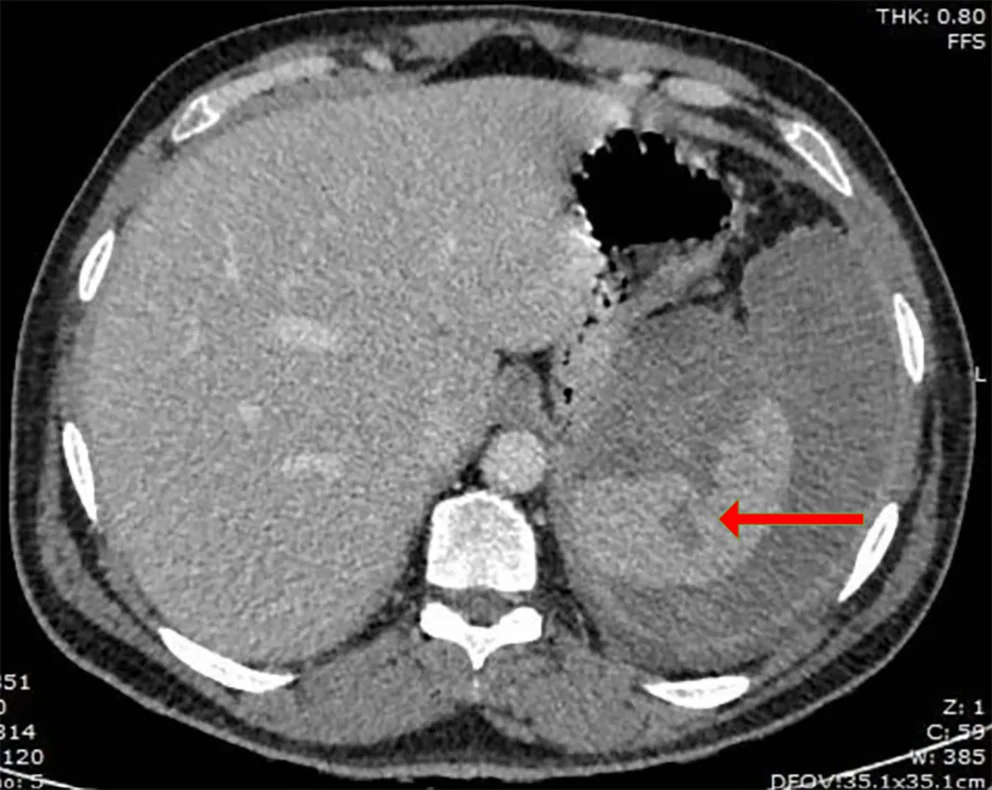
Triphasic CT scan of abdomen showing splenic laceration surrounded by hemoperitoneum.

## 3 Investigations

Pathological findings were compatible with splenic laceration associated with congestion and pseudoaneurysms (Figures 3, 4). On the fifth postoperative day after splenectomy, he developed progressive breathlessness on minimal exertion. Repeat chest x‐ray showed increased left‐sided pleural effusion (Figure 5). Ultrasonography of the chest revealed a minimal, clinically insignificant right‐sided effusion, whereas the left‐sided effusion progressed to a moderate pleural collection without septations or echogenic foci. Around 500 mL of dark green colored pleural fluid was aspirated which was exudative by Light's criteria. Pleural fluid ADA was 9.4 U/L, LDH was 423 U/L, protein 4.6 g/dL, albumin 2.3 g/dL, sugar 102 mg/dL, hematocrit 0%, total cell count of 513 cells/mm^3^ with lymphocyte predominance (86%), and cultures were sterile (Figure 6). Serum proteins were 7.4 g/dL. Postoperative analysis of abdominal drain fluid revealed an amylase concentration of 15 U/L, while concomitant serum amylase levels remained within the reference range (26 U/L). These findings are consistent with the absence of pancreatic fistula, as drain amylase values did not exceed three times the upper limit of normal serum amylase. Pleural fluid analysis demonstrated an amylase concentration of 20 U/L, which is within the normal reference range effectively excluding the diagnosis of pancreaticopleural fistula.

**FIGURE 3:**
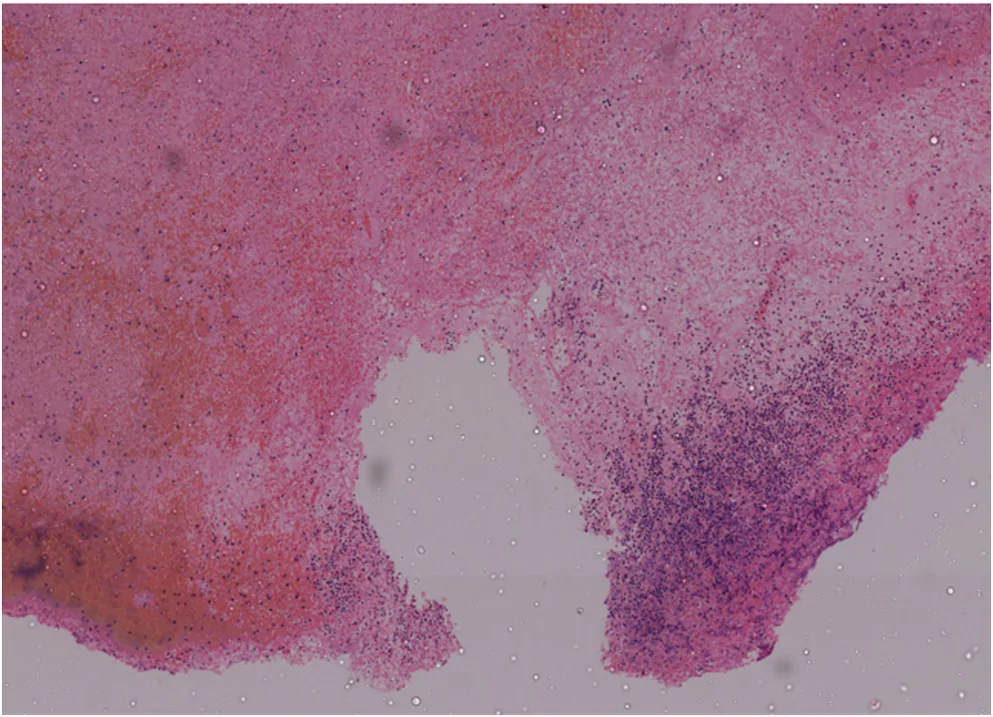
Histopathology of splenic sections showed thick, dense connective tissue capsule with granulation tissue reaction.

**FIGURE 4:**
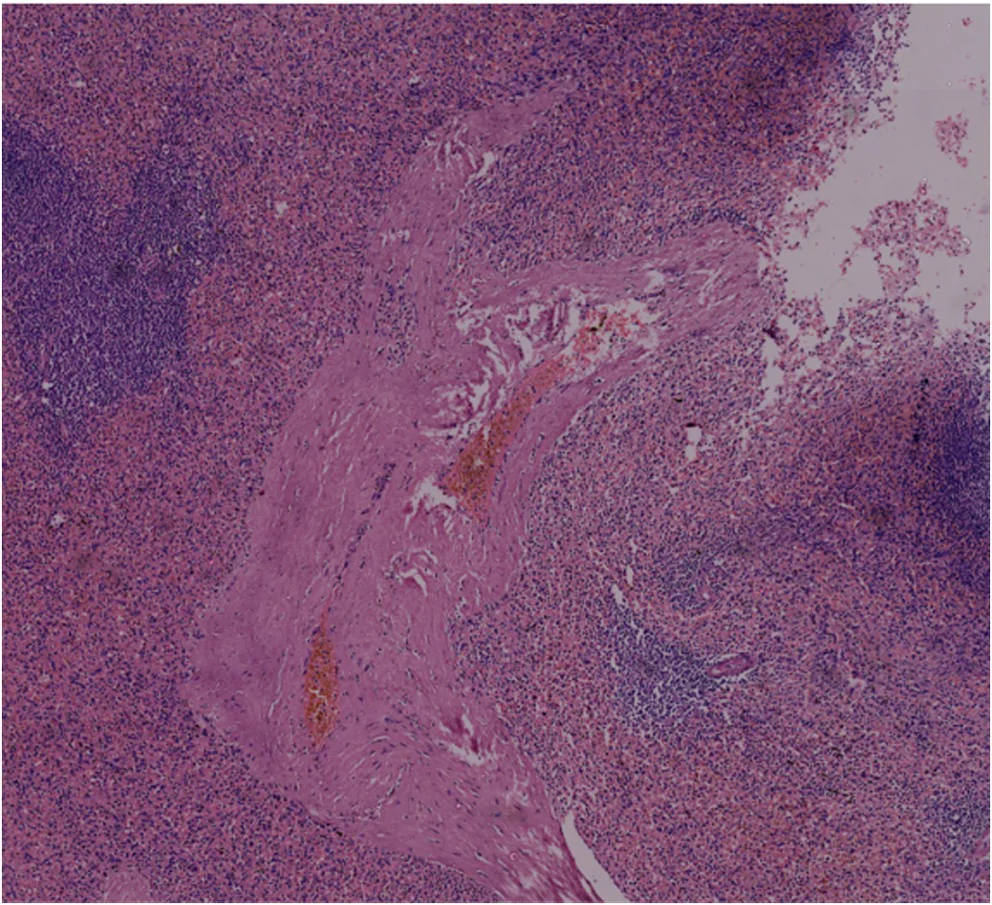
Subcapsular areas showed red pulp expansion by splenic cords, hemorrhage and hemosiderin pigment.

**FIGURE 5:**
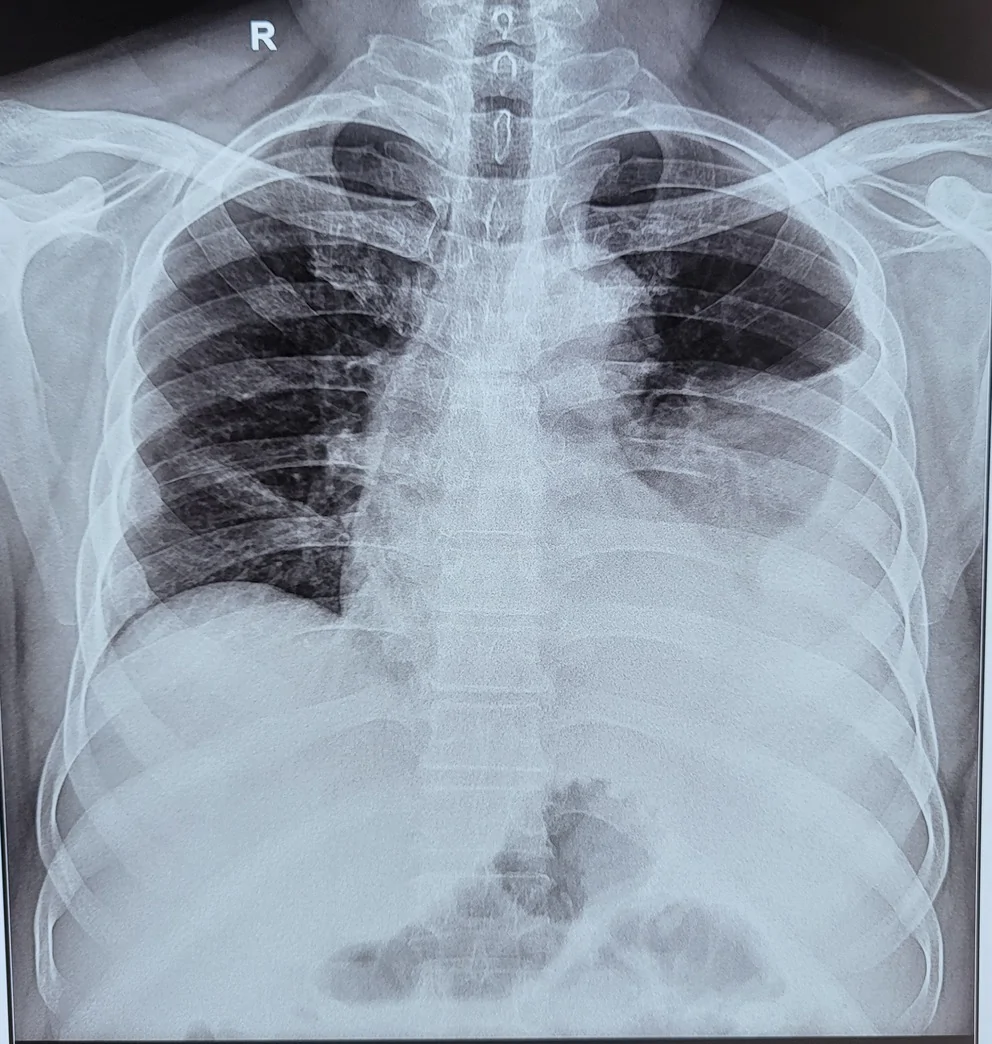
Chest x‐ray showing left moderate pleural effusion on fifth postoperative day.

**FIGURE 6:**
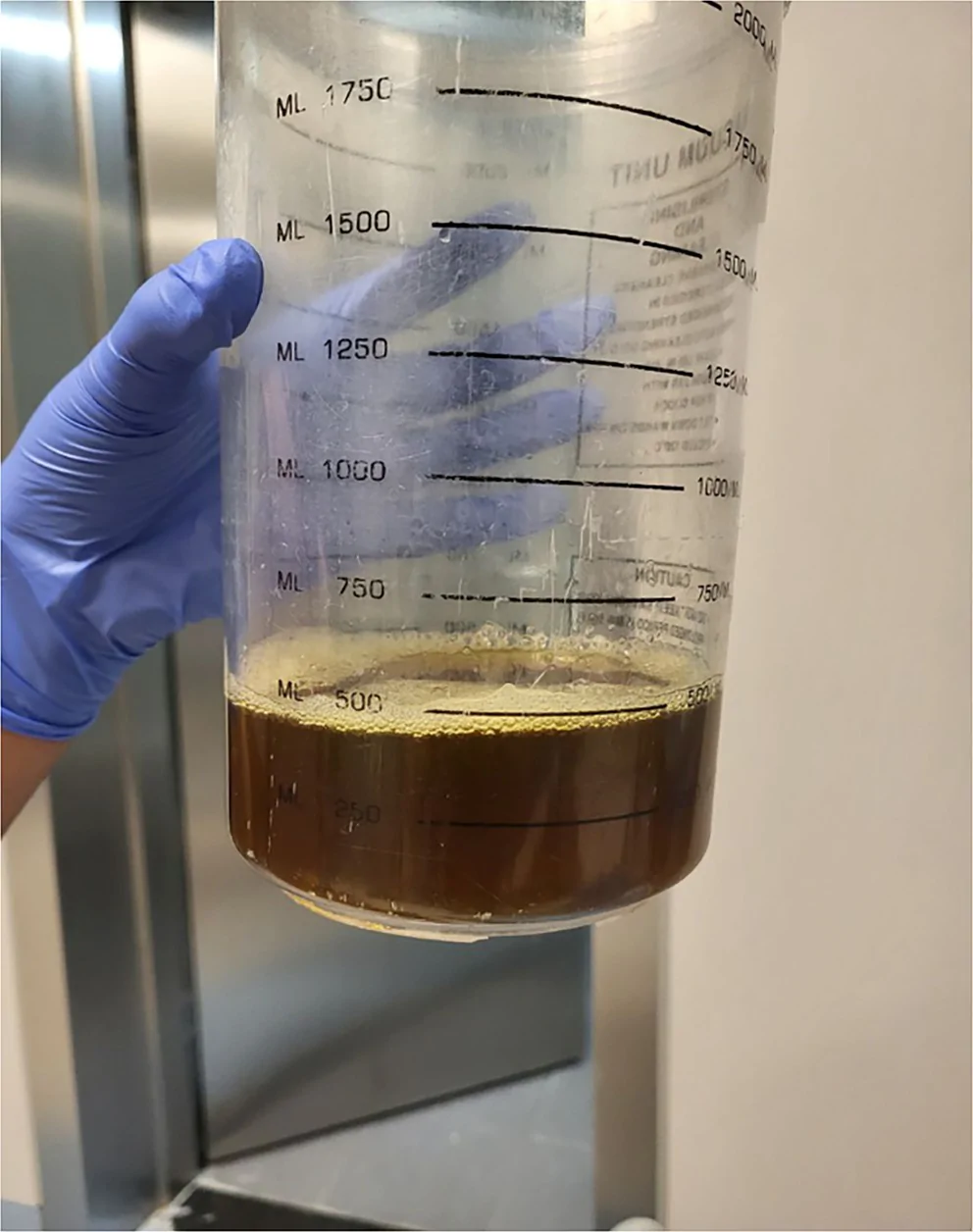
Green colored pleural fluid suggestive of bilothorax.

## 4 Conclusion

In the context of a post‐traumatic presentation, pleural fluid analysis was undertaken due to the distinctive greenish discoloration observed macroscopically. Biochemical evaluation demonstrated a pleural fluid total bilirubin concentration of 2.9 mg/dL and a direct bilirubin concentration of 0.7 mg/dL, in contrast to serum total bilirubin of 0.6 mg/dL and serum direct bilirubin of 0.2 mg/dL. The pleural fluid‐to‐serum total bilirubin ratio exceeded 1, thereby fulfilling the established biochemical criterion for the diagnosis of bilothorax. The patient demonstrated a favorable clinical response following a single therapeutic thoracentesis in conjunction with antimicrobial therapy. The patient received perioperative prophylactic antibiotics with intravenous ceftriaxone, a third‐generation cephalosporin, which was continued for 10 days. The patient demonstrated favorable postoperative recovery and was discharged 2 weeks after surgery. In view of the satisfactory postoperative course, no further interventions or diagnostic evaluations for occult diaphragmatic micro‐injury were done. Serial chest radiographs after discharge obtained at 2‐week, 1‐month, and 3‐months follow‐up revealed complete resolution of the pleural effusion without evidence of recurrence.

To summarize the timeline of events, following blunt abdominal trauma, abdominal ultrasonography in the first week demonstrated splenic injury with hemoperitoneum, accompanied by a mild left pleural effusion on chest radiography. By the third week, triphasic computed tomography of abdomen revealed Grade 4 splenic laceration with moderate hemoperitoneum and trace bilateral pleural effusions. The patient subsequently underwent elective splenectomy in the fourth week. On the fifth postoperative day, chest radiography revealed a moderate left pleural effusion, for which thoracentesis was performed, along with postoperative abdominal drain analysis. Imaging in the sixth and seventh weeks showed complete resolution of pleural effusion, and the patient was discharged. Outpatient evaluations conducted at 2‐week, 1‐month, and 3‐months follow‐up revealed persistently normal chest radiographs, thereby substantiating sustained clinical and radiological recovery. Pneumococcal, meningococcal, and *Haemophilus influenzae* type B vaccines, together with annual influenza immunization, were recommended to the patient to reduce the risk of overwhelming post‐splenectomy infection.

## 5 Discussion

In the systematic literature review conducted by Acharya et al. [3], very few reported cases of bilothorax were left‐sided. Most of the cases of bilothorax occurred in surgical settings. The most common etiology was percutaneous transhepatic biliary drainage (23.47%), followed by trauma, gunshot injuries, or stab wounds involving the right hemithorax or abdomen.

A bilopleural fistula was identified in 51% of patients and was diagnosed using radiologic imaging, video‐assisted thoracoscopic surgery, endoscopic retrograde cholangiopancreatography, or by dye injection into the pleural or abdominal cavity. Only 10% of patients required operative repair of the fistula. Computed tomography identified bilopleural fistulas in merely 25% of cases, whereas hepatobiliary iminodiacetic acid (HIDA) scans or magnetic resonance imaging of the hepatopancreatobiliary system demonstrated superior sensitivity.

Infected bilothorax was observed in approximately 15% of patients, most commonly due to *Escherichia coli* and less frequently *Klebsiella* species, consistent with the predominance of enteric Gram‐negative organisms in biliary infections. This highlights the importance of early recognition and targeted antimicrobial therapy in managing such complications. Overall prognosis was favorable, with 84% of patients achieving clinical improvement. The primary therapeutic approach was drainage of the bilious pleural effusion, most commonly via tube thoracostomy (73.83%), supplemented by serial thoracenteses. Surgical intervention was reserved for cases unresponsive to chest tube drainage, with biliary decompression being the most frequently performed procedure.

Very few cases of left‐sided bilothorax have been documented in the literature. A rare case of spontaneous left‐sided bilothorax in a sickle cell crisis patient has been documented [4]. Another case occurred secondary to cholangiocarcinoma which caused intrahepatic dilatation and a fistulous connection [5]. Other cases were due to trauma from car accidents and nasogastric tube placement, which were complicated by the formation of gastro‐pleural fistulas due to stomach perforation. A rare case of left‐sided bilothorax was described following emergent endoscopic retrograde cholangiopancreatography for acute cholangitis after an episode of hypoxia. The mechanism of bilothorax remained uncertain in the absence of demonstrable fistulization. Proposed explanations included passive transdiaphragmatic migration of bile through microscopic diaphragmatic pores, or drainage of subphrenic biliary collections via the pleuroperitoneal lymphatic channel [6].

We report a unique case of left‐sided moderate bilothorax occurring without biliary tree injury. Bile, synthesized by the liver, consists predominantly of bile salts, phospholipids, cholesterol, and conjugated bilirubin—the terminal product of heme catabolism. In this case, pleural fluid‐to‐serum ratios of total, direct, and indirect bilirubin were all > 1. Additional pleural fluid assays, such as glycocholic acid measurement, may further substantiate the bilious nature of the effusion.

The liver and spleen constitute the principal sites of extravascular hemolysis. The spleen's subdiaphragmatic position adjacent to the left hemidiaphragm predisposes splenic trauma to pleural complications, thereby providing a pathophysiological basis for bilothorax formation analogous to hepatic hydrothorax. In this case, hemolysis from resorption of the splenic hematoma and hemoperitoneum resulted in elevated unconjugated bilirubin, which diffused transdiaphragmatically, culminating in pleural effusion and bilothorax [7].

Enhanced hepatic bilirubin processing and subsequent biliary secretion, followed by secondary translocation into the pleural space, and/or subtle, clinically occult transdiaphragmatic passage of bile in the absence of radiologically demonstrable biliary tract injury cannot be definitively excluded as HIDA scan was not done though CT abdomen showed no biloma.

Early recognition of bilothorax is critical because delayed drainage may result in empyema, loculated effusions, fibrothorax, or respiratory compromise. Prompt drainage of the pleural space, ideally through tube thoracostomy, is recommended to prevent secondary infection. Empiric antibiotics should be initiated early, particularly in patients with risk factors for contamination or infection. Bilothorax should be considered in any new‐onset pleural effusion in patients with a history of hepatobiliary surgery, trauma, malignancy, radiation, or intra‐abdominal infection.

Management primarily involves effective drainage of the bilious effusion, while surgical correction is warranted when a bilopleural fistula is present.

This case emphasizes the importance of maintaining a high index of suspicion for bilothorax, especially in atypical presentations and in the absence of overt hepatobiliary injury. Early diagnosis and intervention are essential to prevent complications and optimize patient outcomes.

## Author Contributions

**Thanuja Reddipilli:** conceptualization, investigation, writing – original draft, writing – review and editing. **Raghava Rao Gandra:** conceptualization, investigation, supervision, validation, writing – original draft, writing – review and editing. **Vishwanath Gella:** conceptualization, formal analysis, supervision, visualization, writing – review and editing. **Deekshitha Ithepalli:** investigation, writing – original draft, writing – review and editing.

## Funding

The authors have nothing to report.

## Consent

The authors declare that written informed consent was obtained for the publication of this manuscript and accompanying images using the consent form provided by the Journal.

## Conflicts of Interest

The authors declare no conflicts of interest.

## Data Availability

The data that support the findings of this study are available from the corresponding author upon reasonable request.
